# Tau antibody isotype induces differential effects following passive immunisation of tau transgenic mice

**DOI:** 10.1186/s40478-021-01147-0

**Published:** 2021-03-12

**Authors:** Rinie Bajracharya, David Brici, Liviu-Gabriel Bodea, Phillip W. Janowicz, Jürgen Götz, Rebecca M. Nisbet

**Affiliations:** 1 Queensland Brain Institute, Clem Jones Centre for Ageing Dementia Research, University of Queensland, St Lucia, Brisbane, QLD 4072 Australia; 2 Translational Research Institute, The University of Queensland, St Lucia, Brisbane, QLD 4072 Australia; 3 Walter and Eliza Hall Institute of Medical Research, 1G Royal Parade Parkville, Melbourne Victoria, 3052 Australia

**Keywords:** Tau, Antibody, Immunotherapy, Microglia, Alzheimer’s disease

## Abstract

One of the main pathological hallmarks of Alzheimer’s disease (AD) is the intraneuronal accumulation of hyperphosphorylated tau. Passive immunotherapy is a promising strategy for the treatment of AD and there are currently a number of tau-specific monoclonal antibodies in clinical trials. A proposed mechanism of action is to engage and clear extracellular, pathogenic forms of tau. This process has been shown in vitro to be facilitated by microglial phagocytosis through interactions between the antibody-tau complex and microglial Fc-receptors. As this interaction is mediated by the conformation of the antibody's Fc domain, this suggests that the antibody isotype may affect the microglial phagocytosis and clearance of tau, and hence, the overall efficacy of tau antibodies. We therefore aimed to directly compare the efficacy of the tau-specific antibody, RN2N, cloned into a murine IgG1/κ framework, which has low affinity Fc-receptor binding, to that cloned into a murine IgG2a/κ framework, which has high affinity Fc-receptor binding. Our results demonstrate, for RN2N, that although enhanced microglial activation via the IgG2a/κ isotype increased extracellular tau phagocytosis in vitro, the IgG1/κ isoform demonstrated enhanced ability to reduce tau pathology and microgliosis following passive immunisation of the P301L tau transgenic pR5 mouse model.

## Introducion

Alzheimer’s disease (AD) is characterised pathologically by the extracellular accumulation of amyloid-β (Aβ) as plaques and the intraneuronal accumulation of hyperphosphorylated tau as neurofibrillary tangles. While Aβ plaques and tau tangles characterize the neuropathology of end-stage AD, it is the small oligomers of Aβ and tau that correlate best with the neurotoxicity driving AD’s early clinical impairments. These small molecules act in concert to exert their effects in such a way that reductions in tau appear to abrogate Aβ-mediated toxicity [1]. This makes tau an attractive therapeutic target [2]. Passive immunotherapy is emerging as a promising strategy for the treatment of these diseases and there are currently a number of tau-specific monoclonal antibodies in clinical trials [3, 4]. Despite tau being predominantly localised within neurons, increasing evidence suggests that pathogenic tau is secreted and able to seed neuronal pathology in a prion-like manner [5, 6]. Therefore, the mechanism of action for some antibodies may not rely on antibody cellular uptake but rather the ability to engage with and clear extracellular pathogenic forms of tau and reduce neuron-to-neuron propagation [7–10]. In support of these proposed mechanisms, tau-specific monoclonal antibodies have been demonstrated to facilitate microglial phagocytosis of extracellular tau in vitro [11], and this process has been shown to require Fcγ-receptor binding and functional lysosomes [12].

Fc receptor binding is mediated by the conformation of the Fc domain of an antibody. Humans have five IgG isotypes (IgG1, IgG2a, IgG2b, IgG3 and IgG4); mice also have five IgG isotypes but these differ in their nomenclature (IgG1, IgG2a, IgG2b, IgG2c, IgG3). These subclasses mediate effector functions differently due to variable specificity and affinity for Fc receptors (FcR), including the intracellular Fc receptor, TRIM21, the neonatal Fc receptor and the family of Fcγ receptors (FcγRIa, FcγRIII, FcγRIV and FcγRIIb) [13]. For example, the murine IgG1 only binds FcγRII and FcγRIII with low affinity, whereas murine IgG2a binds to all receptors in the following order of affinity: FcγRI > FcγRIV > FcγRIII > FcγRIIb [13]. Human IgG1 is the most similar to murine IgG2a as they both have the strongest binding to FcγRs and therefore the greatest ability to activate microglia and induce phagocytosis of the antibody-antigen complex. Human IgG4 on the other hand is most similar to murine IgG1 as they display the weakest ability interact with FcγRs and are poor activators of microglia. This was demonstrated by Adolfsson et al*.* who directly compared an anti-Aβ antibody, MABT, as a human IgG1 isotype and a human IgG4 isotype, containing the same antigen-binding variable domains and with equal binding to Aβ. They showed reduced activation of stress-activated p38MAPK (p38 mitogen-activated protein kinase) in microglia and less release of the proinflammatory cytokine TNFα following treatment with MABT IgG4, compared with the IgG1 isotype [14]. This suggests that whilst a tau-specific monoclonal antibody in a high effector-function isotype may induce the greatest amount of tau phagocytosis, the subsequent release of pro-inflammatory cytokines may be deleterious in vivo.

We therefore aimed to investigate if the IgG isotype specifically affects the therapeutic efficacy of an anti-tau antibody. To achieve this, we cloned the variable domains of our previously characterised RN2N antibody, which is specific for 2 N tau isoforms [15], into both murine IgG1/κ and IgG2a/κ backbones and directly compared their ability to reduce tau. Here we show that despite RN2N IgG2a demonstrating an enhanced ability to clear tau in vitro, RN2N IgG1 demonstrated a superior ability to reduce tau inclusions and microgliosis following passive immunization of tau transgenic pR5 mice.

## Materials and methods

### Antibodies

Primary antibodies used for western blot (WB), immunohistochemistry (IHC) and immunofluorescence (IF) in this study were as follows: Dako Tau (Dako) (WB: 1:10,000), AT180 (pTau231) and AT8 (pTau202/205) (Thermo Fisher) (WB: 1:1000) (IF: 1:500), pTau422 (GeneTex) (WB: 1:1,000) and IBA1 (Wako) (IHC: 1:400). The secondary antibodies used in this study were as follows: Goat anti-mouse Alexa Fluor 488 (Thermo Fisher) (IF: 1:500), polyclonal goat anti-rabbit IgG biotinylated (Dako) (IHC: 1:500), goat anti-mouse IR800 (WB:1:10,000) and goat anti-rabbit IR800 (WB: 1:10,000).

### Antibody generation

RN2N is a 2 N-tau specific antibody raised against the tau peptide TEIPEGITAEEAGI (aa 84–97 of the longest human tau isoform, tau441) [15]. The variable light and variable heavy chains of RN2N were previously cloned into a mouse IgG2a/κ framework [15]. For this study, the variable domains were also cloned into a mouse IgG1/κ framework (mAbXpress vectors kindly provided by the Queensland node of the National Biologics Facility) as previously described [16]. IgG was purified by affinity chromatography then buffer exchanged into 1 × phosphate-buffered saline (PBS) (Protein Expression Facility, The University of Queensland). The concentration of the purified RN2N was determined using a NanoDrop 2000 (Thermo Scientific).

### Recombinant human tau

A cDNA encoding full-length human tau was cloned into the pET-DEST42 vector (Thermo Fisher Scientific) in frame with the C-terminal His6 and V5 tag. Plasmids were transformed into One Shot BL21 bacterial cells (Thermo Fisher Scientific) and recombinant protein expression was induced with 1 mM IPTG. Protein purification was conducted following the protocol outlined in [17].

### Surface plasmon resonance

Surface plasmon resonance measurements were conducted at the Monash Fragment Platform, Monash University, using the Biacore S200 biosensor (GE Healthcare). Biotinylated RN2N was captured on a streptavidin-coated CM5 chip (GE Healthcare). For biotinylation, RN2N IgG1 (29.5 μM) in PBS was added in a 1:1 ratio to EZ-link NHS-LC-LC-biotin (Thermo Fisher Scientific) and incubated at 25 °C for 1 h. The antibody was separated from free, unconjugated biotin by size-exclusion chromatography on a Superdex 200 10/300 GL (GE Healthcare) column equilibrated in PBS. Streptavidin was immobilized on the CM5 chip using amine coupling at 37 °C. Antibody was captured at 25 °C, using a flowrate of 10 µL/min in PBS. Tau binding experiments were run using single-cycle kinetics at 25 °C with the running buffer (12 mM Na_2_HPO_4_, 287 mM NaCl, 2.7 mM KCl, 1.8 mM KH_2_PO_4_, 0.05% Tween-20 pH 7.4). Tau was injected for 120 s at a flow rate of 40 µL/min with a dissociation time of 600 s, using 8 concentrations of tau (1/3 serial dilutions from 0.0128 to 1,000 nM). The data were processed using Biacore S200 Evaluation Software Version 1.0, double referenced against blank injections of buffer and fit to a Steady State Affinity model using report points 4 s before the injection end, with a 5 s window.

### BV2 phagocytosis assay

BV2 cells were cultured in DMEM supplemented with 5% heat-inactivated fetal calf serum. 24 h prior to treatment, cells were plated at 1.25 × 10^5^ cells/well in a 12-well plate and allowed to attach overnight. For microscopy, BV2 cells were plated on Poly-D-Lysine (Sigma) coated glass coverslips 24 h prior to treatment. RN2N IgG and recombinant hTau were conjugated to Alexa Fluor-488 (AF488) (Thermo Fisher) and pHrodo Red protein (Thermo Fisher), respectively, according to the manufacturer’s instructions. hTau-pHrodo (50 nM) was incubated either with or without RN2N IgG-AF488 (50 nM) in Opti-MEM (Thermo Fisher) at room temperature for 1 h. For flow cytometric analysis, proteins were added to plated cells and incubated for 2 h at 37 °C, then cells were trypsinized and resuspended in Hanks’ balanced salt solution (Life Technologies) containing 1% FBS and 1 mM EDTA. Excitation at 488 nM and 560 nM (BD LSR II Analyser) was conducted and data was analysed using FlowJo v10 software (Tree Star). The integrated fluorescence intensity (% Parent x Mean 488 nM intensity) was then calculated. For microscopy, proteins were added to plated cells and incubated for 2 h at 37 °C then cells were fixed with 2% paraformaldehyde (PFA) (Sigma), washed with PBS then counterstained with DAPI (Dako). Cells were washed again with PBS and then mounted onto microscope slides. Imaging was performed using the Axio Imager Z2 (Zeiss).

### sqRT-PCR analysis

For qPCR analysis, protein were added to plated cells and incubated for either 2 or 8 h at 37 °C. Cells were then collected and RNA isolated as described below. Total RNA was isolated from treated BV2 cells using TRIzol reagent (LifeTechnologies), following the manufacturer protocol. Reverse transcription of 200 µg total RNA was performed using SuperScript III reverse transcriptase (LifeTechnologies) and random hexamer primers (LifeTechnologies). Semi-quantitative RT-PCR was performed using 1 µL of the resulting cDNA in a 5 µL total volume containing SSoAdvanced Sybr Green (BioRad) and murine primers (IDT) targeting the genes of interest. For amplification and recording, a CFX384 Touch machine (BioRad) was used, and the results were evaluated using the manufacturer’s software. Amplification specificity was confirmed by melting curve analysis, with quantification performed using the ΔΔCt method.

### Mice

All animal experiments were conducted under the guidelines of the Australian Code of Practice for the Care and Use of Animals for Scientific Purposes and approved by the University of Queensland Animal Ethics Committee (QBI/412/14/NHMRC; QBI/027/12/NHMRC). pR5 mice express 2N4R tau with the P301L mutation under the control of the mThy.1.2 promoter [18].

### In vivo imaging

6 month-old pR5 mice were randomly assigned to one of the following groups: no antibody, RN2N IgG1 and RN2N IgG2a. 24 h prior to treatment, animals were anesthetized with ketamine (100 mg/kg) and xylazine (10 mg/kg), their whole body shaved and residual hair removed using hair removal cream. Immediately prior to treatment, all animals were anesthetized again and prepared as previously described [19]. For the antibody groups, 3 nmol of either RN2N IgG1 or RN2N IgG2a conjugated to AlexaFluor 647 (Life Technologies) was injected retro-orbitally. Mice were kept under 1–2% isoflurane and were scanned 1 h post-treatment using a Bruker In Vivo MS FX Pro optical imaging system with x-ray and a 630 nm excitation and a 700 nm emission filter. Whole animal scans were analyzed using Bruker Molecular Imaging software. An ellipsoid region of interest (ROI) was drawn in the brain of each mouse at every time point post-treatment, with the calibrated unit of radiant efficiency (P/s/mm2) being reported for each ROI. Raw signal was log-transformed to improve Q-Q plot normality.

### Passive immunisation of mice

Four month-old female pR5 mice were randomly assigned to three treatment groups (n = 10 per group): Control, RN2N IgG1 and RN2N IgG2a. Aged-matched wild-type (WT) littermate control mice were also assigned to a WT group for behavioural testing. pR5 mice were injected intraperitoneally once per week for 12 weeks with either 100 μL PBS (control) or 40 mg/kg RN2N IgG1 or RN2N IgG2a. Mice were weighed every week.

### Elevated plus maze

Anxiety-like behavior was assessed in the elevated plus maze (EPM) as previously described [20] with some modifications. Briefly, mice were placed in the central area of the maze (elevated cross-shaped apparatus with a central square, and closed as well as open arms with unprotected edges). The time spent in the three zones over a 5 min period was recorded using an overhead camera with EthovisionXT™ tracking software (Ethovision). The percentage time spent in each arm was calculated. Seven untreated wild-type (WT) littermates were used as controls. Analysis was conducted blinded.

### Open field

The open field test was used to assess general animal activity, exploration and anxiety. The open field consists of a square-shaped box where animal movement, position and speed is monitored by infrared beam breaks that project across the open field along the X, Y and Z axes. Mice were placed in the centre of the box and were allowed to explore for 20 min. The software was set up according to the General Open Field test from the Activity Monitor version 7 manual (MED Associates, Inc.). All data was transmitted to a PC and analysed using Activity Monitor 7 software, SOF-812 (MED Associates, Inc.).

### Tissue processing

24 h after behavioural testing, mice were anaesthetised and transcardially perfused with PBS. Their brains were harvested and the hemispheres separated. The right hemisphere was immersion-fixed in 4% PFA (Sigma) for 24 h, and then embedded in paraffin using a benchtop tissue processor (Leica). Coronal paraffin-embedded brain Sects. (7 and 14 μm thickness) between Bregma − 1.34 mm and − 2.06 mm were obtained using a microtome. The left hemisphere was snap-frozen in liquid nitrogen and stored at − 80 °C until further processing.

### Fractionation of brain tissue

Brains from treated mice were extracted using the RAB/RIPA fractionation method [21]. Brain tissue was homogenized in 6X volume of RAB Buffer (Astral Scientific), followed by centrifugation at 21,000 ×  *g* for 90 min. The supernatant (soluble tau) was collected for western blot analysis. The pellet was resuspended in RIPA buffer (Cell Signalling) then centrifuged at 21,000 × *g* for 90 min. The supernatant (insoluble tau) was collected for western blot analysis. Protein concentrations were measured using a BCA protein assay kit (Thermo Fisher).

### Western blot analysis

15 μg of total protein was electrophoresed on a 10% Tris–glycine SDS-PAGE gel. Proteins were transferred to Immun-Blot Low Fluorescence PVDF membrane (Bio-Rad) using the Transblot Turbo Transfer System (Bio-Rad) and then stained with REVERT^TM^ 700 Total Protein Stain according to the manufacturer’s instructions (LI-COR). Total protein was imaged in the 700 nm channel with the Odyssey FC Imaging System (LI-COR) then membranes were washed and blocked for 30 min in Odyssey^®^ Blocking Buffer (LiCOR). Membranes were incubated in primary antibody overnight at 4 °C by rocking. Membranes were washed with TBS and incubated with the secondary antibody for 30 min at room temperature. Membranes were imaged in the 800 nm channel and fluorescence was quantified using the Image Studio™ Lite software (LI-COR).

### Immunohistochemistry

Mounted brain tissue was first dehydrated in a series of xylene and ethanol washes. Antigen retrieval was conducted in a domestic microwave (850 W) in citrate buffer pH 5.8 for 15 min, followed by cooling at room temperature for 40 min. Paraffin-embedded brain sections between Bregma − 1.34 mm and − 2.06 mm were analysed by immunohistochemistry as described [22], using at least 4 sections per mouse. Briefly, sections were incubated with primary antibodies overnight. For immunofluorescence staining, 7 μm sections were washed and incubated with respective secondary antibodies, then counterstained with DAPI nuclear stain (4′, 6-diamidino-2-phenylindole) (1:5000). For immunohistochemistry staining, 14 μm sections were stained using the nickel-diaminobenzidine (Nickel-DAB) method with no counterstain applied. All images were obtained on an automated slide scanner (Axio Imager Z2, Zeiss) using a Metafer Vslide Scanner program (Metasystems) at 20 × magnification.

### Image analysis

Image quantification and analysis were performed blinded using ImageJ software. For all analyses, a no primary antibody control was used for thresholding. More specifically, for immunofluorescence staining quantification, percentage (%) area positivity for AT8- and AT180-tau were obtained on thresholded images of the amygdala using the area fraction method. For Nickel-DAB staining quantification, Iba1 percentage (%) immunoreactive area (area fraction method) and Iba1-positive average cell sizes were obtained by using the Analyse Particles plugin on thresholded images of an area of the primary somatosensory cortex which was particularly positive for AT180- and AT8-tau in the transgenic mice used in this study. The regions of interest (ROIs) drawn in the Iba1 analyses were kept constant across images. All measurements were noted and averaged over 2–3 sections per mouse.

### Statistical analysis

Statistical analyses were performed with GraphPad Prism 8 software using one-way ANOVA with Tukey’s multiple comparison test. All values are given as the mean ± standard error of the mean (SEM). Outliers were removed using the ROUT method (Q = 1).

## Results

### Antibody isotypes do not affect binding to tau

**Fig. 1:**
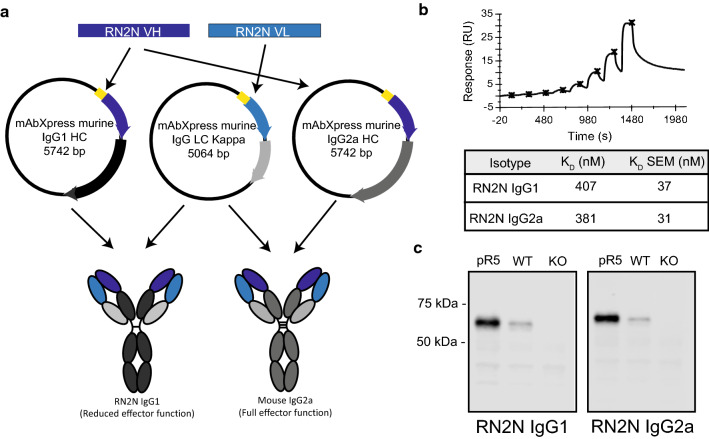
Generation and characterisation of RN2N IgG1 and IgG2a isotypes. **a** Schematic of generation of RN2N IgG1 and IgG2a monoclonal antibodies using mAbXpress IgG vectors. **b** Single-cycle kinetics sensorgram of RN2N IgG1 to full-length human tau and summary table of RN2N IgG1 and RN2N IgG2a binding affinity (K_D_) to full-length human tau determined using surface plasmon resonance. The K_D_ of RN2N IgG1 and RN2N IgG2a was 407 nM and 381 nM, respectively. **c** Western blot of mouse soluble brain extracts probed with RN2N IgG1 and IgG2a antibodies indicating similar binding profiles to tau (pR5 = pR5 tau transgenic mouse; WT = wild-type mouse; KO = tau knock-out mouse)

We have previously cloned the RN2N variable domains into a murine IgG2a/κ backbone (RN2N IgG2a) and determined its binding affinity to recombinant human tau to be 381 nM [23]. To directly compare our tau antibody in different IgG isotype backbones, we cloned the RN2N variable domains into a murine IgG1/κ backbone (RN2N IgG1) and conducted surface plasmon resonance as done in our previous study [23] (Fig. 1a). The binding affinity to human recombinant tau was calculated to be 407 nM (Fig. 1b), demonstrating that RN2N IgG1 and IgG2a have comparable binding affinities to human tau. Furthermore, both RN2N isotypes detected 2 N tau isoforms following western blotting of brain extracts from pR5 tau transgenic and wild-type mice but not in brain extracts of tau knock-out mice (Fig. 1c).

### RN2N IgG2a treatment demonstrates enhanced phagocytosis of tau-antibody complex by BV2 microglia cells compared to RN2N IgG1 treated cells

**Fig. 2:**
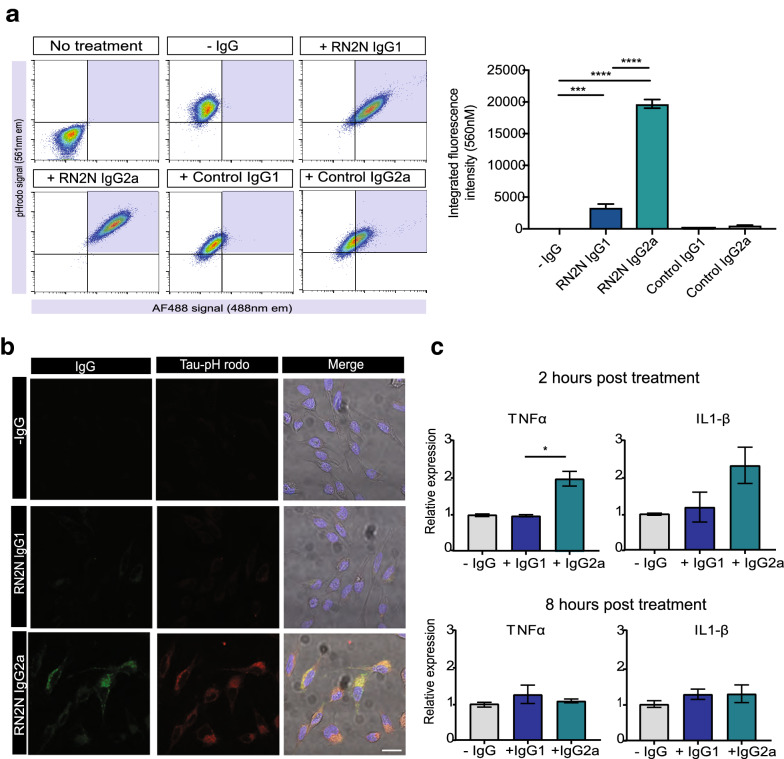
**a** RN2N IgG2a treatment demonstrates enhanced BV2 microglial cell phagocytosis of tau and increased transcription of pro-inflammatory cytokines. Flow cytometric analysis and quantification of tau-pHrodo fluorescence in BV2 microglial cells treated with either RN2N IgG1, RN2N IgG2a, control IgG1 or control IgG2a (***p < 0.001, ****p < 0.0001; *n* = 3 for each group). **b** Representative microscopy images of BV2 microglial cells treated with RN2N and human recombinant tau showing phagocytosis of RN2N IgG (green) and internalized Tau-pHrodo (red) complexes. Nuclei were stained with DAPI in blue. Scale bar = 20 μm. **c** BV2 microglial cells were treated with recombinant human tau with or without RN2N IgG, and TNFα and IL1-β transcription was analysed using sqRT-PCR at 2 or 8 h post-treatment (*p < 0.05; *n* = 3 for each group)

To investigate the ability of the RN2N IgG isotypes to activate microglial phagocytosis of tau, BV2 microglial cells were treated with recombinant human tau conjugated to pH-rodo in the absence or presence of RN2N IgG. FACS analysis revealed that tau on its own, and when incubated with the control IgGs, was phagocytosed to a small extent (Fig. 2a). This was significantly increased, however, when tau was incubated with RN2N IgG1, and even more so when incubated with RN2N IgG2a (Fig. 2a). This demonstrates that the binding of RN2N to tau specifically stimulates microglial phagocytosis and that RN2N IgG2a is more efficient at mediating tau phagocytosis compared to RN2N IgG1. This was also observed by microscopy, which showed increased phagocytosis of tau in the presence of RN2N IgG2a, compared to in the absence of IgG, and only minimal phagocytosis of tau in the presence of RN2N IgG1 (Fig. 2b).

### RN2N IgG2a, but not RN2N IgG1, induces pro-inflammatory cytokine transcription following treatment

Microglial stimulation of the Fc receptors can lead to pro-inflammatory cytokine release. Therefore, to determine if tau-specific antibody isotypes resulted in different levels of pro-inflammatory cytokine release, sqRT-PCR was conducted on cells treated with or without the two IgG formats for 2 or 8 h. At 2 h, transcription of the pro-inflammatory cytokine, tumor necrosis factor alpha (TNFα), was significantly increased in BV2 cells treated with tau in the presence of RN2N IgG2a, but not in the RN2N IgG1 treated group (Fig. 2c). However, this effect was absent at 8 h (Fig. 2c). Similarly, at 2 h, transcription of the pro-inflammatory cytokine interleukin 1 beta (IL1-β), showed a trend towards an increase in the RN2N IgG2a treated cells compared to those treated with tau alone or in the presence of RN2N IgG1, and this effect was also absent at 8 h (Fig. 2c). These findings suggest that the engagement of tau by RN2N IgG2a, but not RN2N IgG1, and the subsequent stimulation of microglia, result in the increased acute production of pro-inflammatory cytokines.

### Treatment with RN2N IgG2a, but not IgG1, induces a disinhibition-like behaviour in pR5 mice

**Fig. 3:**
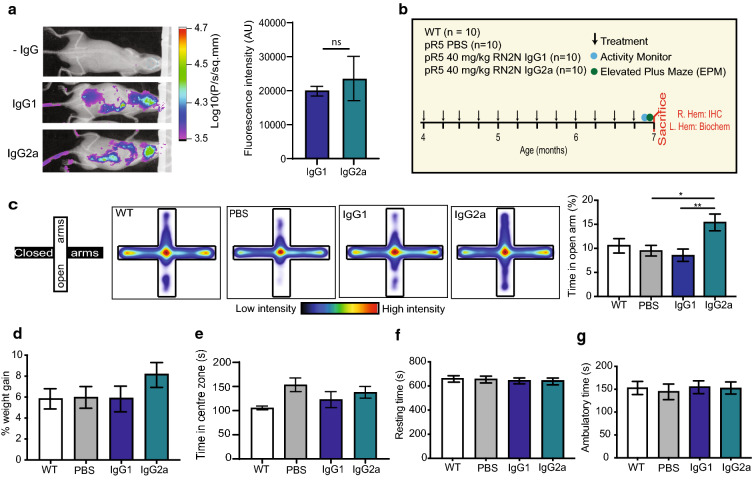
RN2N IgG2a treated mice demonstrate an increased disinhibition-like behaviour following passive immunization of pR5 mice. **a** RN2N IgG isotype does not affect brain delivery in vivo following intravenous administration of Alexa-Fluor 647-conjugated RN2N. Representative images of Alexa Fluor 647-conjugated RN2N administered pR5 mice at 60 min post-treatment using a Bruker In Vivo MS FX Pro optical imaging system and quantification showing the mean fluorescence counts within the outlined region of interest were reported as logarithmic scale following the subtraction of the mean of control (− IgG) (*n* = 3 for each group). **b** Schematic of passive immunization treatment and behavioural analysis schedule in pR5 mice. **c** Elevated plus maze (EPM) results showing that mice treated with RN2N IgG2a spend more time in the open arms compared to the control and RN2N IgG1 treated mice. Schematic of EPM arena with its open and closed arms. Mean positional heat map within the EPM for each treatment group and quantification of % time spent in open arms over the duration of the test (*n* = 10 for each group). **d** Percentage (%) weight gain of treatment groups from start of treatment compared to end of treatment (*n* = 10 for each group). **e**–**g** Activity monitor (open field) showing no differences in treatment groups in **e** time spent in the centre zone, **f** resting time and **g** ambulatory time (*n* = 10 for each group). *p < 0.05, **p < 0.01

Our in vitro studies have demonstrated that RN2N in the high FcR-binding IgG2a format enhances microglial phagocytosis of tau compared to the low FcR-binding IgG1 format. This process, however, results in a transient increase in expression of pro-inflammatory cytokines which might reduce the therapeutic efficacy of RN2N in the IgG2a format. We therefore aimed to determine the ability of both RN2N IgG formats to reduce tau pathology in P301L tau transgenic pR5 mice which express the longest brain tau isoform, 2N4R, and are characterized by progressive tau pathology predominantly in the amygdala and to a lesser extent in the hippocampus and cortex [18]. Prior to treating mice, we sought to compare the delivery of the different RN2N isotypes to the brains of these mice (Fig. 3a). In vivo whole-body imaging 1 h post intravenous delivery of fluorescently-conjugated RN2N IgG revealed that both RN2N isotypes were detected in the brains of mice (Fig. 3a), with no significant difference between IgG1 and IgG2a following quantification of the fluorescence intensity in the brains of the mice (Fig. 3a). This suggests that any differences in efficacy following passive immunisation of pR5 mice is not likely due to differences in brain uptake. We then went on to treat pR5 mice once per week for twelve weeks with either vehicle only (PBS), RN2N IgG1 or RN2N IgG2a (Fig. 3b). Upon completion of the treatment, the behaviour of the pR5 mice was investigated using the elevated plus maze (EPM) (Fig. 3c). We have previously shown that pR5 mice have increased anxiety-like behaviour as characterised by a reduction in the time spent in the open arm of the EPM [15]. In the current study, however, there was no difference in the time spent in the open arm between the WT and pR5 mice, suggesting a loss of this behavioural phenotype in this cohort of pR5 mice possibly due to genetic drifting (Fig. 3c). Interestingly, however, mice treated with RN2N IgG2a, demonstrated a significant increase in the time spent in the open arms of the EPM compared to the control pR5 mice and mice treated with RN2N IgG1 (Fig. 3c), suggesting an increase in disinhibition-like behaviour in these mice. Furthermore, pR5 mice treated with IgG2a, but not IgG1, showed a trend towards an increase in weight gain over the course of the treatment (Fig. 3d). In the open field, however, there was no differences observed between the treatment groups in the time spent in the centre zone (Fig. 3e), resting time (Fig. 3f) or ambulatory time (Fig. 3g).

### Phosphorylated soluble and insoluble total tau levels are reduced independent of antibody isotype following passive immunization of pR5 mice

**Fig. 4:**
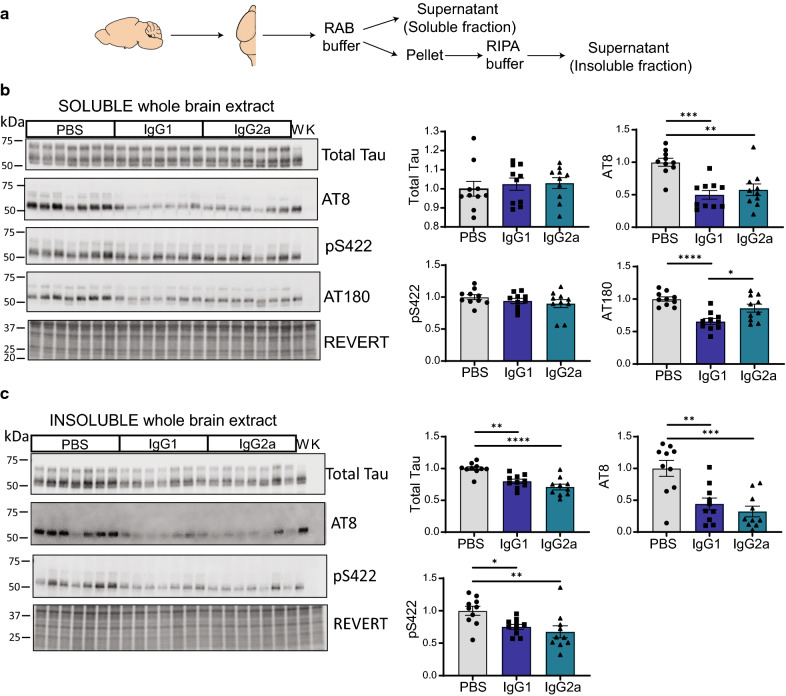
RN2N IgG1 and IgG2a treatments reduce total insoluble and phosphorylated tau in pR5 mice. **a** Schematic of RAB/RIPA brain fractionation protocol to obtain soluble (RAB) and insoluble (RIPA) fractions of whole brain lysate. **b** Representative images of soluble pR5 brain lysate probed with anti-tau antibodies as indicated (W = brain homogenate from wild-type mice; K = brain homogenate from *MAPT* knock-out mice) and quantification (*n* = 10 for each group). **c** Representative images of insoluble pR5 brain extracts probed with anti-tau antibodies as indicated and quantification (*n* = 10 for each group). *p < 0.05, **p < 0.01, ***p < 0.001, ****p < 0.0001

To investigate the effect the different RN2N isotypes had on tau pathology, the brains of treated mice were dissected and analysed by western blotting following sequential protein extraction (Fig. 4a). In the RAB-soluble fractions, western blotting with the human tau-specific antibody, Dako Tau, demonstrated no differences in total tau in mice treated with RN2N compared to control treated mice (Fig. 4b). The phospho-tau specific antibodies AT8 and AT180, however, demonstrated a significant reduction in tau phosphorylated at these epitopes following treatment with both RN2N isotypes compared to control treated mice. Interestingly, there was a significant difference in the amount of AT180 immunoreactive tau in the RN2N treated groups with the IgG1 isotype reducing AT180 immunoreactive tau to a greater extent than IgG2a. On the other hand, investigation of serine 422-phosphorylated tau revealed no significant difference in the RN2N treated groups compared to control (Fig. 4b). In the RIPA fraction, however, which contains insoluble, pathogenic species of tau, a reduction in total tau was observed in RN2N treated mice, regardless of isotype, compared to control mice (Fig. 4c). This was also seen with the phosphorylated-tau specific antibodies, AT8 and anti-serine 422. It is important to note that AT180 immunoreactive tau in the RIPA fraction was below the detection limit of the assay. Taken together, these data demonstrate the ability of both RN2N IgG1 and IgG2a to reduce tau phosphorylation and reduce the formation of insoluble tau species.

### RN2N IgG1, but not RN2N IgG2a, reduces tau inclusions in the amygdala following passive immunization of pR5 mice

**Fig. 5:**
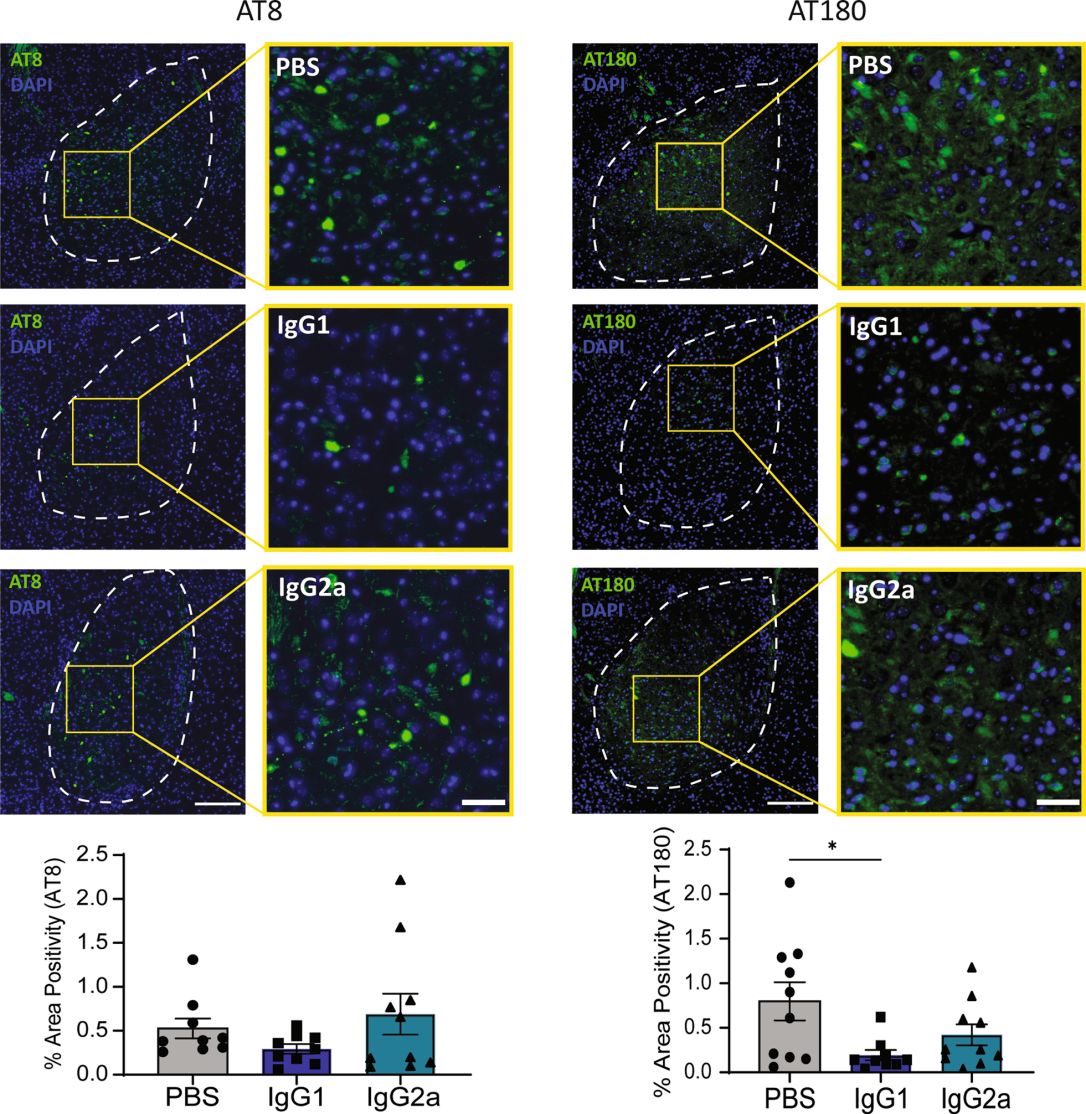
RN2N IgG1 treatment reduces AT180-positive tau inclusions in the amygdala of pR5 mice. **a** Representative images of AT8 immunoreactivity observed in the amygdala of pR5 mice in each treatment group and quantification of % area positivity of AT8-positive cells in the amygdala (PBS: *n* = 9; IgG: *n* = 10; IgG2a: *n* = 10). **b** Representative images of AT180 immunoreactivity observed in the amygdala of pR5 mice in each treatment group and quantification of % area positivity of AT180-positive cells in the amygdala (PBS: *n* = 10; IgG1: *n* = 9; IgG2a: *n* = 10). There was a significant reduction in % area positivity of AT180 in the amygdala in mice treated with IgG1 compared to mice treated with PBS. *p ≤ 0.05. Scale bars: left panel, 200 μm; right panel, 50 μm

To determine the effect of RN2N treatment on the formation of tau inclusions, phosphorylated tau positive neurons in the amygdala, the main brain region that is affected in pR5 mice at this age [18], were counted following immunofluorescence analysis. Treatment with RN2N IgG1, but not IgG2a, significantly reduced the number of AT180 positive inclusions compared to control treated mice (Fig. 5). Quantification of AT8-positive tau inclusions in this region, however, did not show a significant difference in the RN2N-treated groups compared to control mice, although there was a trend towards a reduction in the IgG1-treated mice compared to control mice (Fig. 5).

### RN2N IgG1, but not RN2N IgG2a, reduces microgliosis following passive immunization of pR5 mice

**Fig. 6:**
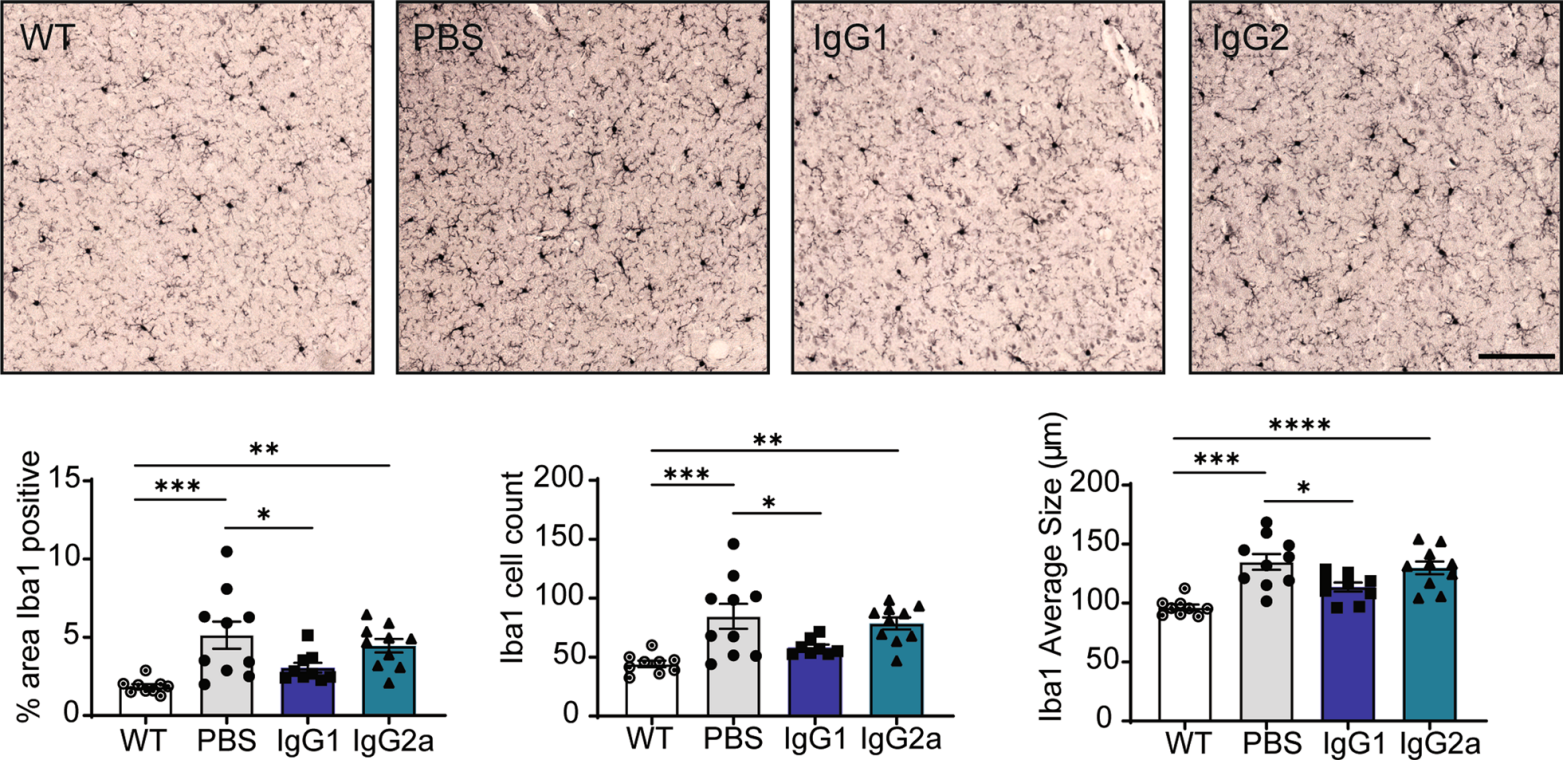
RN2N IgG1 reduces microgliosis following passive immunization. Iba1 labelling of cortical brain sections of WT and pR5 mice in three treatment groups. PBS and IgG2a treated pR5 mice demonstrate a significant increase in the Iba1-positive percentage area, cell count and average cell size compared to WT mice. Treatment of pR5 mice with IgG1 restores Iba1-positive percentage area, cell count, and average cell size to WT levels (WT: *n* = 9; PBS: *n* = 10: IgG1: *n* = 9; IgG2a: *n* = 10). **p ≤ 0.01, ***p ≤ 0.001, ****p ≤ 0.0001. Scale bar: 100 μm

Tau transgenic mice are known to exhibit increased microgliosis and this can be demonstrated by staining for Iba1, a cytoplasmic microglial marker [24]. In the pR5 mice, we observed an increase in the number of Iba1-positive microglia in the cortex compared to WT littermate controls (Fig. 6). To determine the effect of antibody isotype on microgliosis following passive immunisation, brain sections of treated mice were labelled with Iba1. Total cortical microglial surface area, cell count and average cell body size were all significantly reduced in the mice treated with RN2N IgG1, but not RN2N IgG2a, compared to the PBS treated mice, suggesting that only IgG1 treatment can reduce microgliosis observed in tau transgenic mice (Fig. 6).

## Discussion

Tau-targeting passive immunotherapy is a promising strategy for the treatment of AD, with a number of monoclonal antibodies currently in clinical trials, delivered as either a humanized IgG1 or IgG4 isotype [25]. As a direct comparison of a tau monoclonal antibody as a high-effector function isotype and low-effector function isotype has not previously been conducted, we therefore aimed to directly compare the in vitro and in vivo efficacy of our RN2N tau-specific antibody in two murine IgG-isotypes: (i) the high-affinity FcR-binding IgG2a (equivalent to human IgG1), and (ii) the low-affinity FcR-binding IgG1 (equivalent to human IgG4).

Here, we demonstrate that RN2N, in both the IgG1 and IgG2a format, reduced insoluble tau in the P301L tau transgenic pR5 mouse model. The reduction in tau pathology was more pronounced in the IgG1-treated group, however, with only IgG1 reducing phospho-tau positive inclusions in the amygdala. Furthermore, only IgG1 treatment was able to reduce microgliosis. This is despite the IgG2a isotype demonstrating enhanced phagocytosis of tau in vitro. Treatment with the IgG2a isotype in vitro, however, increased the secretion of pro-inflammatory cytokines following tau engagement, suggesting that enhanced phagocytosis and/or enhanced microglial activation with the subsequent release of pro-inflammatory cytokines may be able to induce indirect disease toxicity and overshadow therapeutic effects of a tau-specific antibody. This is consistent with the work of Lee et al*.* who demonstrated that although a full-effector antibody and an effector-less antibody reduced the accumulation of tau pathology following treatment of P301L tau transgenic mice, only the effector-less tau antibody protected neurons from tau-induced toxicity in vitro [26]. In addition, recent studies have shown that microglia are able to take up and secrete tau seeds capable of seeding pathology for neuron-to-neuron propagation [27]. Furthermore, tau-mediated activation of the microglial inflammasome was shown to increase the activity of caspase-1 and downstream IL-1β release, thereby inducing tau pathology [28].

Beyond the effect of the RN2N antibodies on tau pathology, we also observed a surprising behavioural phenotype in the RN2N IgG2a treated mice. In the EPM, the RN2N IgG2a treatment group demonstrated a disinhibition-like behaviour, demonstrated by an increased time spent in the open arms of the EPM, that was not observed in the RN2N IgG1 treated group. In the open field, however, all treatment groups similarly avoided the centre zone, indicating similar anxiety levels. This highlights a disinhibition-like behaviour rather than reduced anxiety levels. Disinhibition-like behaviour has been previously reported in a number of mutant tau transgenic mouse models when compared to non-transgenic mice in the EPM [29–34]. In addition, a disinhibition-like behaviour has been observed in a mouse model of traumatic brain injury which is characterised by both tau pathology and microgliosis following injury [35]. This suggests that pathology caused by tau accumulation or possibly microglial activation, particularly in the amygdala, may contribute to impulsivity and disinhibition-like behaviour in mice. Therefore, rather than improving therapeutic outcomes, treatment with the IgG2a isotype may actually worsen mouse behaviour to recapitulate the behaviour observed in human disease.

Whilst enhanced microglial activation in the IgG2a treated mice and subsequent release of pro-inflammatory cytokines is one explanation for the differences we observed between RN2N IgG1 and RN2N IgG2a in vivo, the physicochemical properties of the Fc domains is another explanation for these differences. A study by Congdon et al. showed that neuronal uptake of tau antibodies and their efficacy strongly depends on antibody charge, with an increase in antibody charge inhibiting the antibody’s ability to be internalized by neurons [36]. RN2N IgG1 has a net charge of 0.4, whereas RN2N IgG2a has one of 3.3, suggesting that RN2N IgG1 may have an enhanced ability to be internalised into neurons and target intraneuronal tau. To date, however, we have not observed any intraneuronal uptake of RN2N IgG in our experiments.

Taken together, our study supports the growing body of evidence that demonstrates that high tau-antibody effector function is not required for antibody efficacy following passive immunization and that a robust microglial response in vivo may be deleterious. Therefore, low-effector function tau antibody isotypes, such as humanized IgG4, and engineered antibodies that lack effector-function all together, such as scFvs [15], Fabs and effector-less IgGs [26], may be safer and more effective alternatives for clinical development.

## Abbreviations

AD: Alzheimer’s disease
IgG: Immunoglobulin
FcR: Fc receptor
scFv: Single-chain variable fragment
TNFα: Tumor necrosis factor alpha
IL1-β: Interleukin 1 beta
Iba1: Ionized calcium-binding adaptor protein-1

## Declarations

### Data accessibility statement

The datasets generated during and/or analysed during the current study are available from the corresponding author on reasonable request.
